# Virus taxonomy: the database of the International Committee on Taxonomy of Viruses

**DOI:** 10.1093/nar/gkaf1159

**Published:** 2025-11-26

**Authors:** Eden J Black, C Steve Powell, Donald M Dempsey, R Curtis Hendrickson, Logan R Mims, Elliot J Lefkowitz

**Affiliations:** Department of Microbiology, University of Alabama at Birmingham, Birmingham, AL 35294, United States; Department of Microbiology, University of Alabama at Birmingham, Birmingham, AL 35294, United States; Department of Microbiology, University of Alabama at Birmingham, Birmingham, AL 35294, United States; Department of Microbiology, University of Alabama at Birmingham, Birmingham, AL 35294, United States; Department of Microbiology, University of Alabama at Birmingham, Birmingham, AL 35294, United States; Department of Microbiology, University of Alabama at Birmingham, Birmingham, AL 35294, United States

## Abstract

Taxonomic classification underlies all biological science and is the basis for comparative analysis of biological organisms and therefore our understanding of life. The International Committee on Taxonomy of Viruses (ICTV) develops the official taxonomy for all viruses. To ensure that the taxonomic data, associated metadata, and analytical tools used to search and visualize those data are easily accessible, the ICTV maintains a comprehensive database and website that provides these resources to the scientific community and the interested public. This report describes the extensive enhancements made to these resources since our first Nucleic Acids Research Database Issue publication in 2018. These enhancements have focused on improvements to the computational infrastructure supporting the database, website, and tools; expanding the information available on the taxonomy and the viruses classified by that taxonomy; enhancing existing and developing new tools to access, search, and display taxonomic data; expanding the available methods and links used to access the taxonomic and associated data; and providing outreach and training opportunities to our users to ensure that these resources are useful and used. The data and tools provided through this effort are available from the ICTV website at https://ictv.global.

## Graphical Abstract

**Graphical Abstract:**
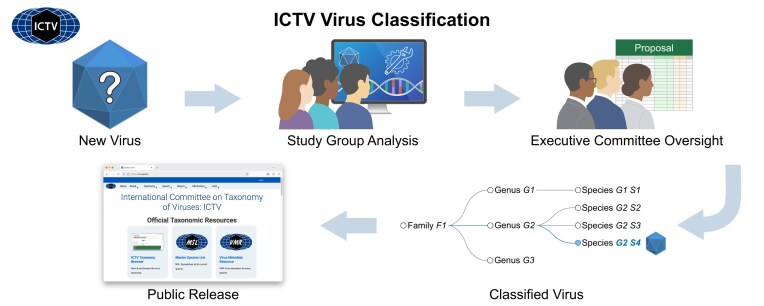

## Introduction

The classification of biological organisms requires a logical framework that can be superimposed upon the natural world [1, 2]. The science of taxonomy provides such a framework through the classification and naming of biological entities [3]. Taxonomic classification results in organisms with similar properties organized into groups and placed into hierarchies that define different levels of shared similarities. These hierarchies are the taxonomic ranks (species, genus, family, order, class, phylum, kingdom) defined by the classification process [4]. The virus taxonomy adds an additional rank, realm, as the highest level in the taxonomy. The biological descriptors that define the taxonomic rank within a particular taxonomic lineage form a unique set of characteristics that identify the organisms classified into that rank and serve, in essence, as a barcode that can be used to identify other potential members of that taxon [5–8]. Critically, the process of taxonomic classification provides a starting point—an initial reference—guiding all subsequent research designed to enhance our understanding of the organisms of interest. By comparing the properties of the organisms classified into one taxon (e.g. species) with those of another, we can begin to delineate the important similarities and differences between the two taxa. For example, by classifying a newly isolated virus into an existing species, we might be able to infer additional properties of the new virus by using the properties of the viruses already classified into that species as a starting point for additional research [9, 10]. In this manner, a reiterative process of classification, inference, testing, and potentially reclassification is established that helps to expand our knowledge of the role of the newly isolated viruses in the ecosystem of interest.

Importantly, taxonomic classification is not a static endeavor. Organisms are reclassified as we learn more about their properties and the properties of related organisms, and newly discovered organisms also need to be classified [11]. Therefore, the organizations that are responsible for overseeing the taxonomy of different biological domains operate under a set of guidelines that determine the rules, policies, procedures, nomenclature, and timelines under which their taxonomy is updated. The organizations that have these responsibilities include the International Commission on Zoological Nomenclature (ICZN; https://www.iczn.org) [12] and the International Committee on Systematics of Prokaryotes (ICSP; https://www.the-icsp.org) [13], which are responsible for the nomenclature of animal and bacterial taxa, respectively.

The organization responsible for the taxonomy of viruses was established in 1966 and named the International Committee on Nomenclature of Viruses [14–16]. It was renamed the International Committee on Taxonomy of Viruses (ICTV) in 1975 [17]. In contrast to other taxonomic organizations such as the ICZN and ICSP, the ICTV is responsible for both classification and nomenclature. The ICTV is charged by the Virology Division of the International Union of Microbiological Societies (IUMS) with developing, refining, and maintaining the official, universal virus taxonomy [18]. The ICTV, through its Executive Committee (EC), Subcommittees, and Study Groups, develops both the guidelines for the classification of viruses as well as the guidelines for naming of the resultant taxa. The rules governing the ICTV and its operations are defined by the ICTV Statutes (https://ictv.global/about/statutes), while the rules for creating and naming virus taxa are provided in the International Code of Virus Classification and Nomenclature (https://ictv.global/about/code). The Statutes and Code were last updated in March 2025.

Taxonomic classification and naming within the ICTV are overseen by the EC, which is composed of a president, vice president, 3 secretaries, 7 subcommittee (SC) chairs, and 11 elected members (https://ictv.global/members/ec-members). For classification, viruses are grouped according to the type of host they infect and the molecular composition of the virus genome, with each group being the responsibility of one of the SCs (https://ictv.global/about/organization). Each SC has Study Groups (SGs), usually one for each taxonomic family, whose responsibilities are to determine the unique set of properties that can be used to distinguish the viruses belonging to one species, genus, family, or higher taxonomic rank from those of the other viruses classified into a corresponding rank. These properties comprise the demarcation criteria that are the basis for taxonomic classification. The SGs then prepare and evaluate proposals for making changes to existing taxa and creating new taxa. There are presently 138 SGs covering all currently classified viruses (https://ictv.global/study-groups).

The ICTV also welcomes taxonomic proposals from any interested individual. Proposal template files and instructions are available at https://ictv.global/taxonomy/templates. Once the relevant files are completed, they should be emailed to the appropriate SC chair. Proposals are then reviewed by the relevant SG and the EC, and any comments or questions are sent to the authors for a response.

Taxonomic proposals for viruses without an established SG are submitted to the SC chair, who oversees the group of viruses covered by the proposal (see https://ictv.global/study-groups). The SC chair will then submit the proposal to the SG with the most relevant expertise. For example, the family *Arteriviridae* is handled by the Nidovirales SG. Any questions regarding the preparation or submission of proposals, including the demarcation criteria used to support classification, should be sent to the relevant SC chair.

### Current issues in virology

The field of virology has undergone significant changes in the past few decades, including several challenges that impact taxonomic classification. These changes and challenges must be addressed by the ICTV so that the taxonomy remains current and relevant. For example, there have been advancements in metagenomics through the bulk sequencing of mixed populations of viruses and other organisms collected from environmental or host samples [19–21]. These sequence data can then be assembled into apparently complete virus genomes that comprise a large number of previously unidentified and therefore unclassified isolates [22–24]. At the same time, there has been a shift by the ICTV to use properties directly derived from the complete virus genome sequences (e.g. nucleotide or amino acid sequence similarity, phylogenetic analysis, open reading frames, gene content, and synteny) as the primary basis for classification rather than physical properties of the virion or other phenotypic characteristics [25]. This approach to classification obviates the need to isolate, grow, and study a physical virus particle and use its properties as the basis for classification. Therefore, metagenomic sequencing and the use of those sequences by the ICTV to support classification represent a profound shift in the requirements necessary to define new taxa. This shift has required that the ICTV update existing demarcation criteria, adopt new techniques and new tools to process the sequence data, and establish robust and scalable databases and query tools to handle the large datasets generated by these processes [26, 27]. SGs establish and update, as necessary, the demarcation criteria for the virus taxa under their purview. The bioinformatics tools used for classification are also described as part of the demarcation criteria and are specific to each family. The necessity for creating new or changing existing demarcation criteria will usually occur when new viruses are discovered, expanding the properties of the viruses to be classified. These criteria may also be updated when new tools become available that have been shown to do a better job classifying a family’s viruses into distinct taxa.

Other challenges derive from the emergence and reemergence of viral diseases and the potential for the rapid spread of new viral pathogens as they are introduced into new hosts, evolving in real-time to better adapt to those hosts. Through the procedures outlined above that support taxonomic classification, the ICTV can assist in the worldwide response to new outbreaks of virus disease by helping to determine the classification of novel pathogens, their relationship to known viruses, and what inferences about form, function, and action might reasonably be made between known and unknown viruses based on these comparisons.

## Recent developments

To respond to these needs, we submitted and received a grant award from the U.S. National Institute of Allergy and Infectious Diseases of the National Institutes of Health to modernize and extend the robustness and functionality of the ICTV data infrastructure. These enhancements to the ICTV database and website have proceeded on the basis of five goals: (i) modernize the information technology infrastructure used to provide all services, moving to an open-source, cloud-based platform; (ii) expand the information provided to understand the properties of the classified viruses and the process used to support classification; (iii) develop easy-to-use software tools to search the taxonomy and associated metadata database and visualize the results; (iv) provide methods to make all of the data programmatically accessible through a variety of computational systems and data repositories; and (v) provide outreach and training assistance to ensure that users can easily access and understand the available information. In our previous report, published in the NAR Database Issues [28], we described the data, tools, and other resources developed prior to receipt of the NIH award. In this updated report, we describe the significant enhancements that have been made over the subsequent eight years.

### Infrastructure

The ICTV will celebrate its 60th anniversary in 2026, and the current ICTV database and website had their beginnings 18 years ago [16]. These long-term commitments to virus taxonomy emphasize the need to ensure a stable and reliable, secure, scalable, and sustainable infrastructure that supports the objective of communicating the products of ICTV activities to interested individuals and groups around the world. Previously, the ICTV information technology (IT) infrastructure was locally housed, proprietary, and expensive to purchase and maintain. Our goal was to develop a modern, easily maintained system, built on an open-source foundation, that would meet the needs of the ICTV for the foreseeable future. These goals have been accomplished, and the components of the upgraded systems are provided in Table 1.

**Table 1.** ICTV Information Technology Systems

Infrastructure component	Implementation	
	Past (proprietary)	New (cloud-based, open-source)
Database, web, application, and development servers	Individually purchased hardware servers; housed locally; backed up to local tape storage systems	Amazon Web Services (AWS)-based cloud infrastructure using AWS Elastic Compute Cloud (EC2) instances; Elastic Block Storage; AWS snapshot backups (https://aws.amazon.com)
Operating system	Microsoft Windows Server	Ubuntu Server (https://ubuntu.com)
Web server	Microsoft IIS	Apache (https://www.apache.org)
Database server	Microsoft SQL Server	MariaDb (https://mariadb.org)
Content management framework	Telligent Community	Drupal (https://drupal.org)

The past and current information technology systems used to provide all database, website, and application resources for the ICTV.

### Information

Our mission, as stated in the ICTV Statutes, is to communicate the decisions reached concerning the classification of viruses and nomenclature of virus taxa to virologists (and other interested groups and individuals) and to maintain an official index of approved names for virus taxa. Therefore, the provision of information on virus taxonomy represents our primary goal. This is accomplished by storing all data and metadata in a relational database and establishing a web-based communication infrastructure. The resources described below have been developed to provide our users with the tools required to access, search, and utilize that information.

#### Taxonomy browsing

Once taxonomic decisions are ratified by the ICTV membership (https://ictv.global/members; “About” menu > “Membership”), they become official, and it is then the responsibility of the ICTV to provide these decisions to the scientific community. This is accomplished through the ICTV database, which stores the taxonomy structure, taxon names, and associated metadata. This information is then available online through the ICTV website as a searchable and expandable table through the taxonomy browser (https://ictv.global/taxonomy; “Taxonomy” menu > “Taxonomy Browser”) as well as a downloadable spreadsheet, the Master Species List (MSL; https://ictv.global/msl, https://doi.org/10.5281/zenodo.15042255; “Taxonomy” menu > “Master Species Lists”). A second spreadsheet, the Virus Metadata Resource (VMR; https://ictv.global/vmr, https://doi.org/10.5281/zenodo.15042309; “Taxonomy” menu > “Virus Metadata Resource”), containing a list of virus exemplars for each virus species, is also available. These exemplars provide an example of a well-characterized, sequenced virus isolate for each species. Entries include the virus name, isolate designation, suggested abbreviation, GenBank accession number(s), segment names, genome composition, and host or sample source.

Proposals for new or updated taxonomy that have been submitted and approved are available from the website (https://ictv.global/files/proposals/approved; “Taxonomy” menu > “Proposals” > “Approved Proposals”). Pending proposals submitted, but not yet officially approved, are also available (https://ictv.global/files/proposals/pending; “Taxonomy” menu > “Proposals” > “Pending Proposals”). Proposal template files, along with instructions for filling out and submitting new taxonomic proposals, are available for download (https://ictv.global/taxonomy/templates; “Taxonomy” menu > “Proposal Template Files”).

#### Taxon history

**Figure 1.**
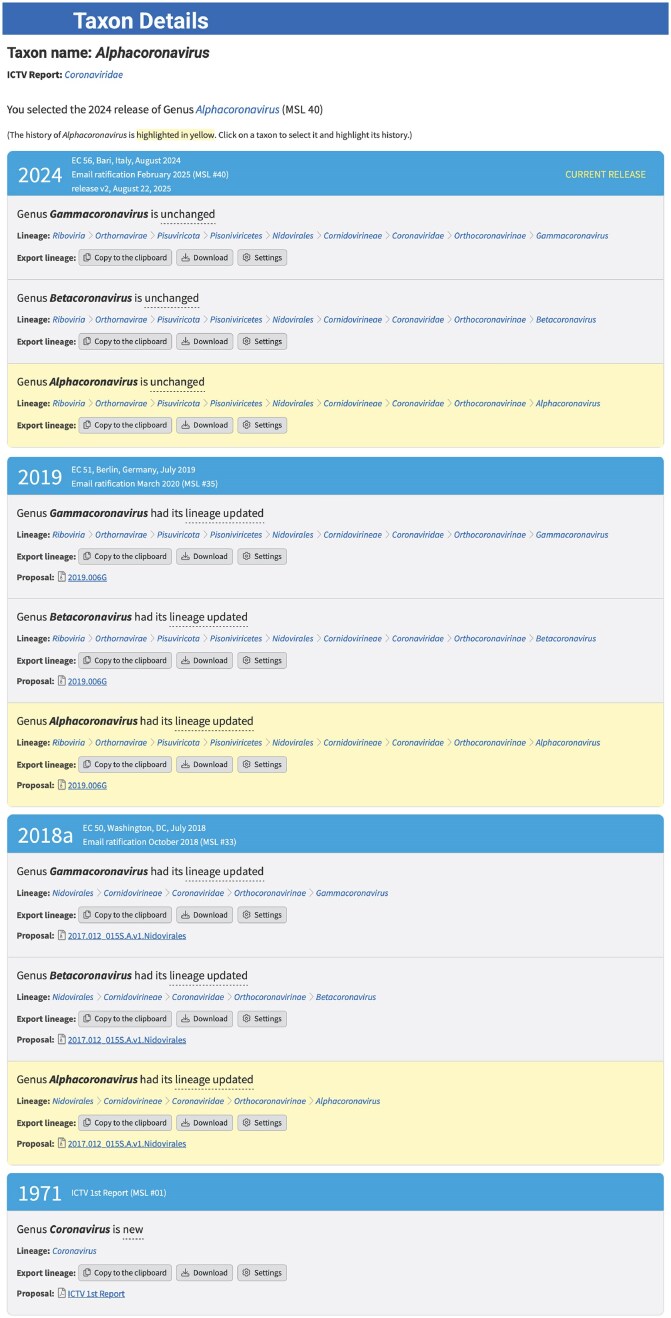
Taxon Details page. An example web page that displays the complete history of a particular taxon and other associated metadata (https://ictv.global/taxonomy/taxondetails?taxnode_id=202401848&taxon_name=Alphacoronavirus).

**Figure 2.**
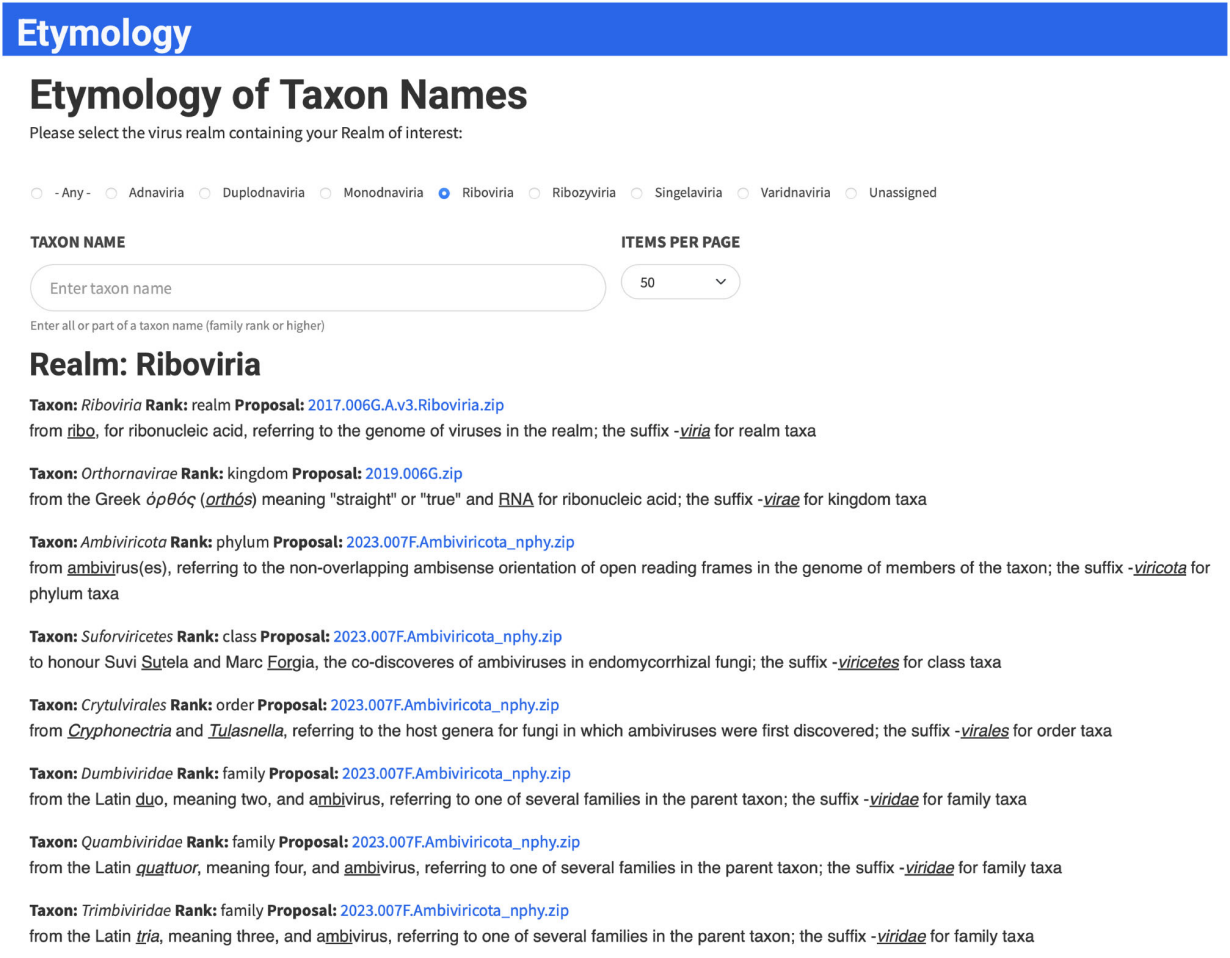
Taxon Name Etymology. Web page that provides descriptions of the origins of ICTV taxon names starting at the rank of realm and going down to the rank of family (https://ictv.global/taxonomy/etymology).

Links from individual taxa in the taxonomy browser, as well as in the MSL and VMR spreadsheets, connect to a taxon details page that provides detailed information on each taxon, including a complete history of all updates to the taxon over time (Fig. 1). The history includes all changes to the taxon since it was first approved and links to the approved proposal documents explaining and justifying each change. The following changes are allowed: new, abolished, renamed, moved, lineage updated, merged, split, promoted, or demoted. Information on the etymological origins of the taxon name is also provided for all ranks at the family level and higher. A separate table listing name etymologies for a user-selected list of taxa is also available (https://ictv.global/taxonomy/etymology; “Taxonomy” menu > “Taxon Name Etymology”; Fig. 2).

#### ICTV Report

**Figure 3.**
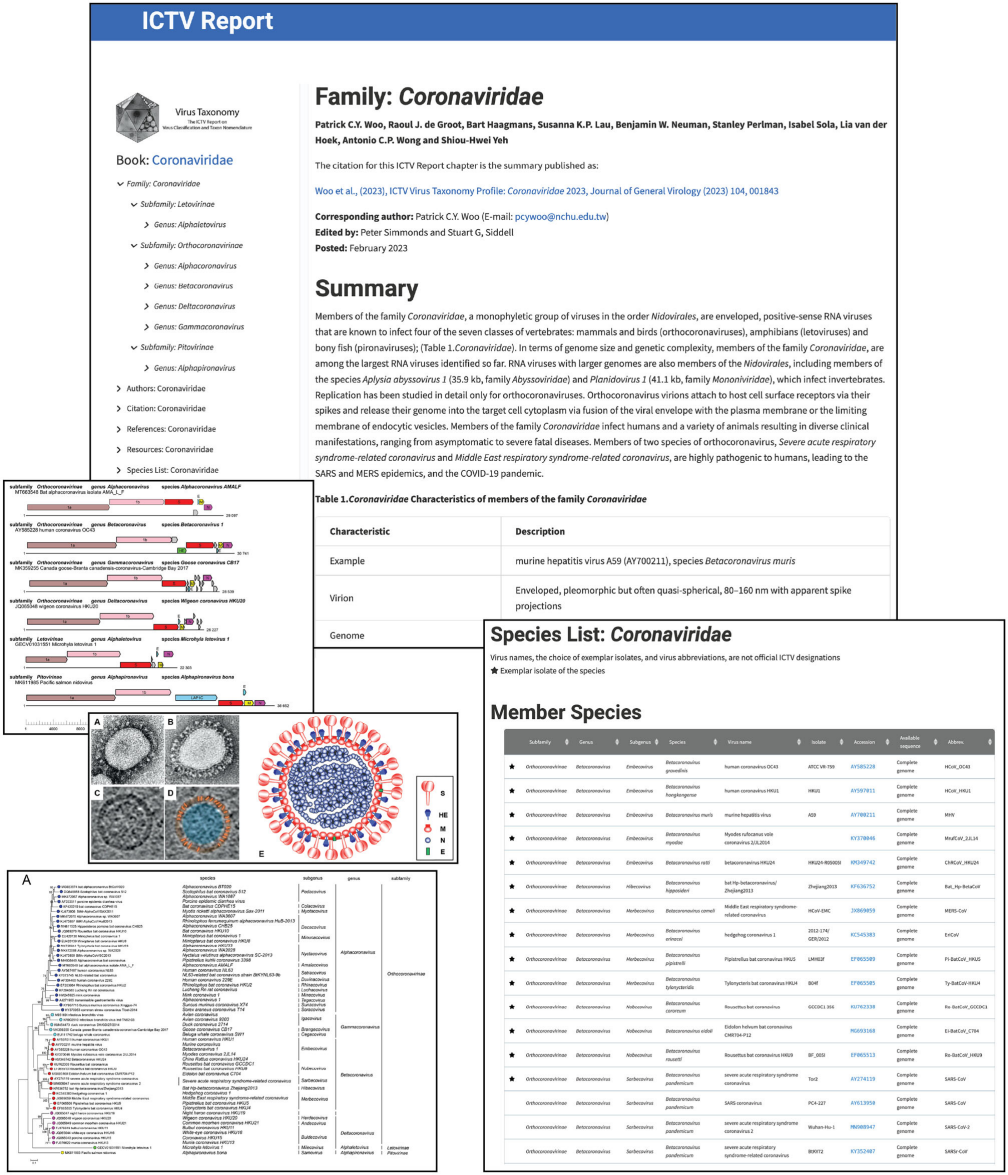
ICTV Report Chapter. Example of the web page components that comprise the Family section of an ICTV report chapter (https://ictv.global/report/genome).

In-depth information on the viruses classified into a particular taxonomic family, as well as the family’s constituent genera and species, is provided by the ICTV Report on Virus Classification and Nomenclature (https://ictv.global/report; “Report” menu > “Current Report Chapters”). These online report chapters replace the printed hard copy versions of the Report that had previously been published every several years and consist of nine volumes that were released from 1971 to 2011 (https://ictv.global/report/about; “Report” menu > “About the ICTV Report”) [14, 17, 18, 29–34]. The layout and content of the current report were substantially upgraded as a part of the infrastructure migration project, as described in Table 1. Figure 3 provides screenshots from the *Coronaviridae* Report chapter (https://ictv.global/report/coronaviridae; “Report” menu > “Current Report Chapters”), showing elements that are typically present in most chapters, including descriptive information, a table of virus properties, virion diagrams, genome maps, phylogenetic trees, and a table of species with links to the genomic sequence accession number(s) of the exemplar virus(es) for each species. Descriptive information is also provided for the subfamily and genus members of the family.

**Figure 4.**
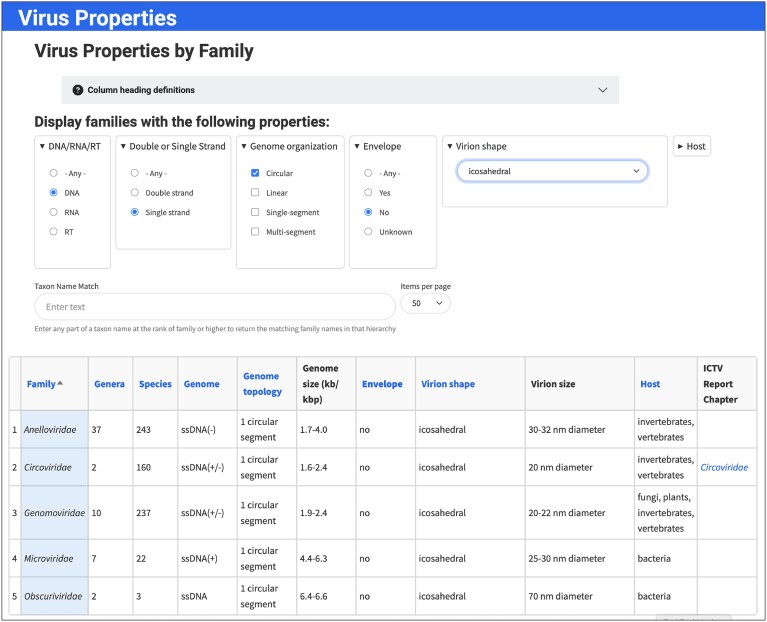
Virus Properties Table. Web page displaying various physical properties of the viruses classified into a particular family. Drop-down selection lists are used to filter the virus list to those families containing viruses with the indicated properties (https://ictv.global/virus-properties).

**Figure 5.**
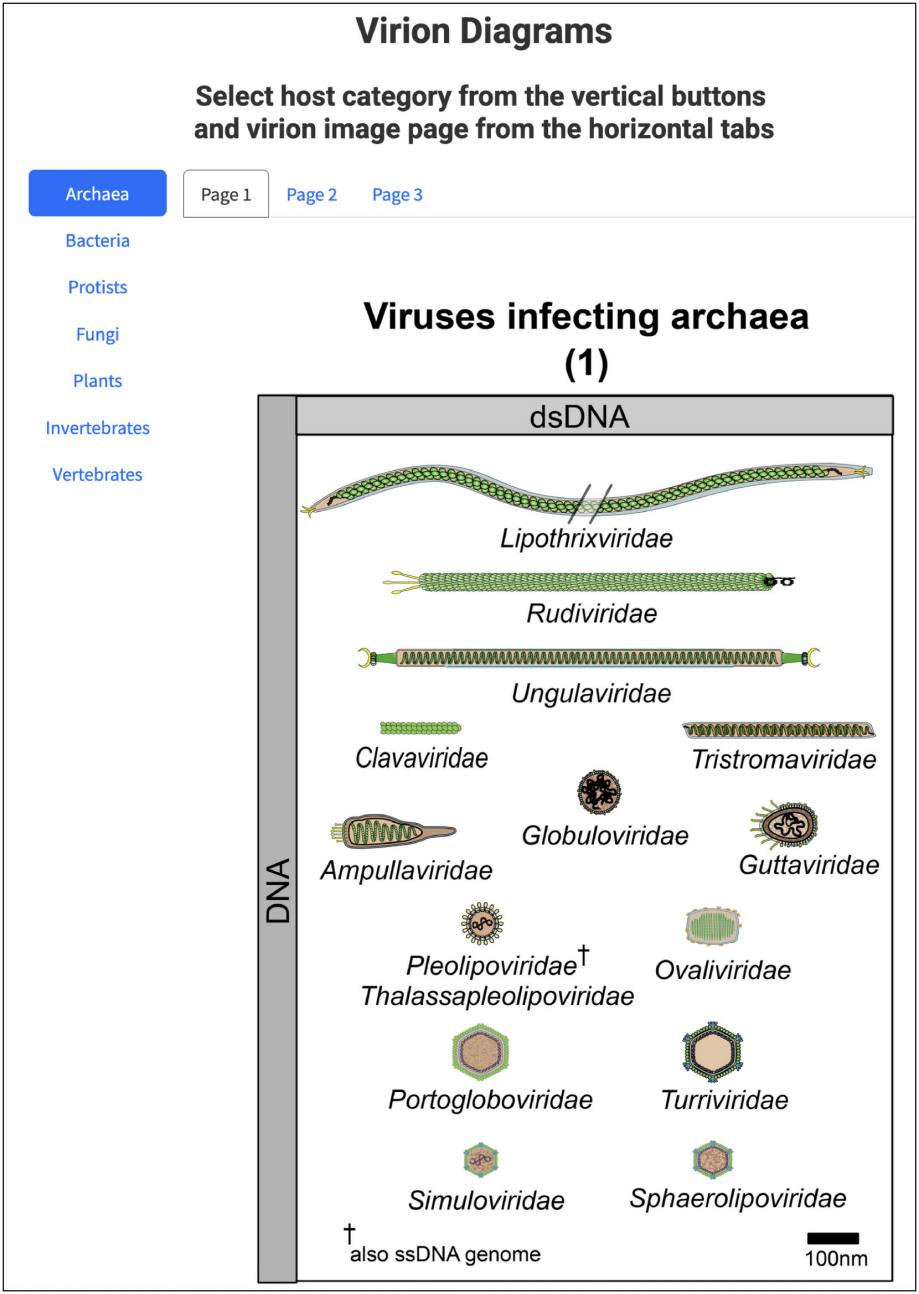
Virion Diagrams. Diagrams of virion morphology arranged by host and genome type at the rank of family (https://ictv.global/report/chapter/information/information/diagrams); diagrams are courtesy of ViralZone (https://viralzone.expasy.org/).

The report also provides data that can be used for direct comparative analysis of the properties of the classified viruses. These data include the Virus Properties table that displays physical and biological properties of the exemplar viruses within each family, with filterable and sortable options for taxon, genome composition and topology, envelope, virion shape, and host (https://ictv.global/virus-properties; “Report” menu > “Virus Properties”; Fig. 4). In addition, diagrams of the exemplar virions for a family are also provided (https://ictv.global/virion-diagrams; “Report” menu > “Virion Diagrams”; Fig. 5). These diagrams are courtesy of ViralZone (https://viralzone.expasy.org/) [35].

### Tools

A variety of tools are provided on the ICTV website to enhance a user’s ability to search for a taxon of interest and visualize its taxonomic classification. New tools, recently made available, are the Visual Taxonomy Browser, Find the Species search tool, and TaxaBLAST.

#### Visual Taxonomy Browser

**Figure 6.**
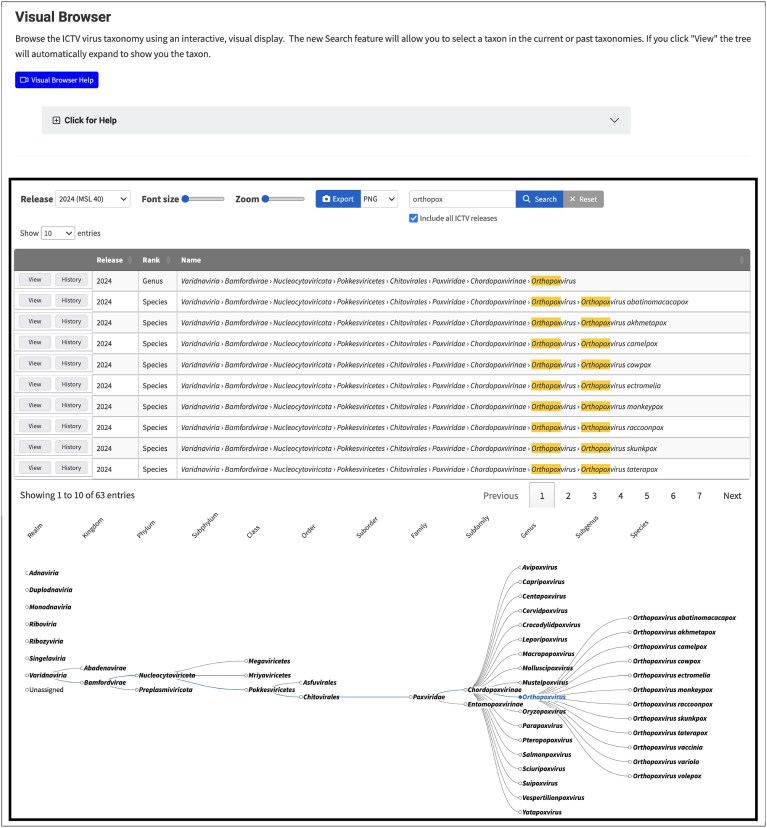
Visual Taxonomy Browser. A browser to visualize the taxonomic lineage of a selected virus taxon using an interactive, visual display and vector graphic export options for creating publishable figures (https://ictv.global/taxonomy/visual-browser).

The visual browser (https://ictv.global/visual-browser; “Taxonomy” menu > “Visual Taxonomy Browser”; Fig. 6) provides a graphical representation of the taxonomy. In contrast to the original browser, which displays the taxonomy in a tabular format with expanding rows, the visual browser displays the taxonomy in an animated, branching tree. This format, although presenting the same information as the original browser, allows for better visualization of the relationships between different taxonomic lineages. Other features of the visual browser include a drop-down list from which one may select a historical MSL release as the basis for a search, font size and zoom controls, and the ability to export images as PNG, SVG, or PDF files for use in publications.

#### Find the SPECIES

**Figure 7.**
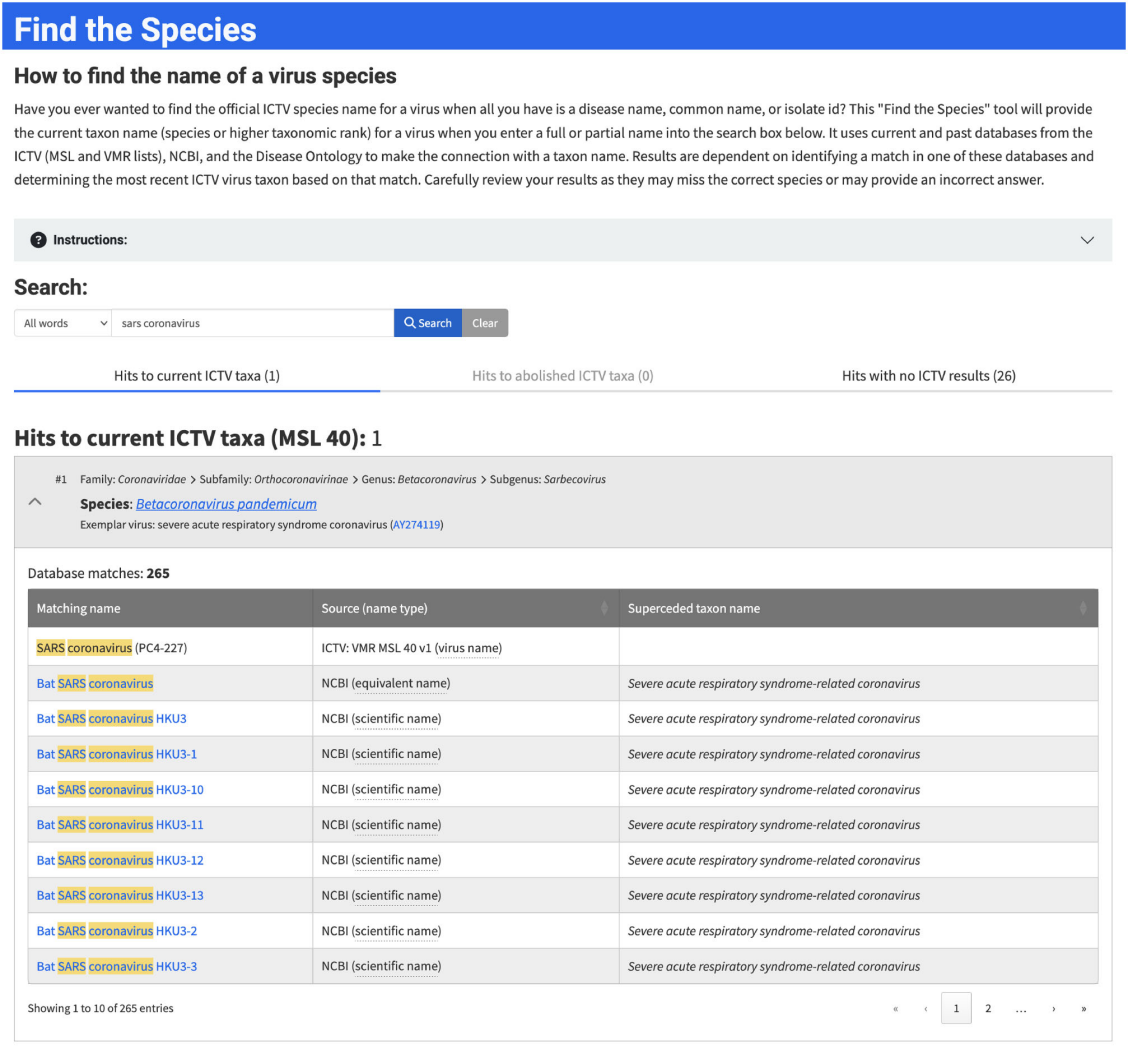
Find the Species. A tool that will provide the current taxon name (species or higher taxonomic rank) for a virus when a full, partial, common, or disease name is entered into the search box (https://ictv.global/search/find_the_species).

One of the most frequently requested features for the ICTV website has been a tool that would allow users to input different names that have been associated with a virus and return the current, official species name. Find the Species is a new tool that provides this functionality (https://ictv.global/find-the-species; “Search” menu > “Find the Species”; Fig. 7). The official ICTV species name for a virus will be returned from a query that matches all or part of a virus or virus isolate name, a past species name, or a disease name. Name synonyms, common names, acronyms, abbreviations, etc. can also be used. Selectable search parameters include finding exact matches, matches to all words or any words entered (in any order), or substring matches. Find the Species uses current and past databases from the ICTV (MSL and VMR lists), National Center for Biotechnology Information (NCBI), and the Disease Ontology to make the connection between the name entered as the search query and an ICTV taxon name. Results are dependent on identifying a match in one of these databases and determining the most recent ICTV virus taxon based on that match. Since its release, Find the Species has become the third-most accessed page of the ICTV website.

#### TaxaBLAST

When a new virus is isolated, one of the first questions that arises is which already described virus is most closely related to the newly discovered virus. In essence, the discoverer of the new virus wants to know the taxonomic placement of their virus. The virus could be classified as a member of an existing species, in which case its complete taxonomic hierarchy is already defined. Or it could represent a new species or a new higher-level taxonomic rank. If this is the case, a proposal should be submitted to the ICTV proposing that a new taxon or taxonomic hierarchy be created. The properties that are used to distinguish one taxon from another are the demarcation criteria that are defined by each ICTV SG for each taxonomic rank within the family covered by the SG. The demarcation criteria can be complex, and the process of determining taxonomic placement can require the use of multiple, not always easy to use, bioinformatics tools. To provide an approximate answer to the question of taxonomic placement, we developed TaxaBLAST (https://ictv.global/search/TaxaBLAST; “Search” menu > “TaxaBLAST”) that provides a quick assessment of the taxonomic placement for a newly isolated virus based on its partial or whole genome sequence. It should not be used as justification for classification in a taxonomy proposal, but it can provide the user with useful information to guide subsequent analysis.

**Figure 8.**
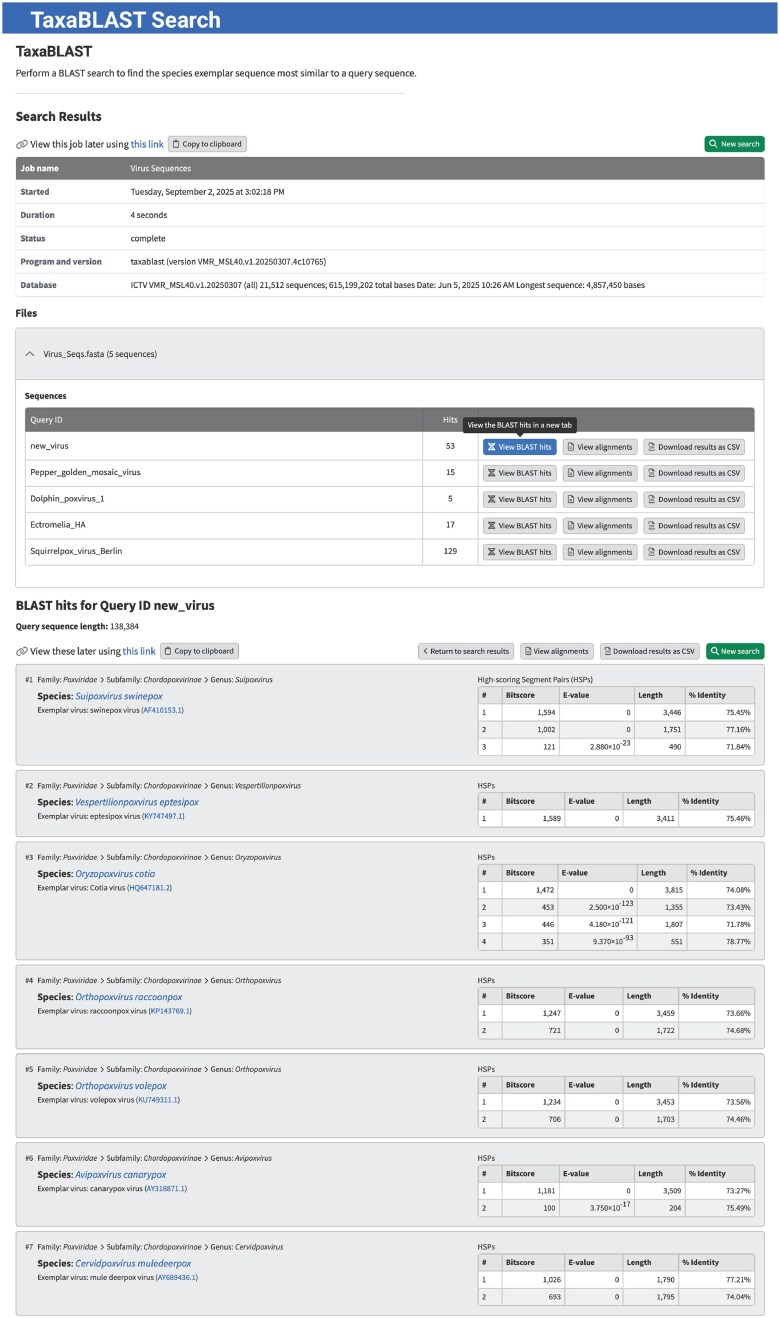
TaxaBLAST. A tool that uses NCBI BLAST to search for the ICTV virus exemplar sequence(s) that is the most similar to a user provided query sequence (https://ictv.global/search/TaxaBLAST).

TaxaBLAST uses a BLAST [36, 37] database that is created from the genomic sequences of all virus exemplars listed in the VMR. When a user enters their query nucleotide sequence into the TaxaBLAST interface, a blastn search using default parameters is run comparing this sequence with the exemplar database (Fig. 8). The application returns a summary of the BLAST hits along with an HTML file containing the sequence alignments and a CSV file containing the BLAST hit statistics. By examining the list of hits returned and their BLAST bit scores, a user is provided with an indication of the species (and higher taxon) with the highest sequence similarity to their virus sequence.

### Accessibility

ICTV data are made available in a variety of formats for multiple audiences: interactive web visualizations and downloadable spreadsheets and documents (the MSL and VMR) for the typical user, and raw, software-consumable data via web services and database exports for the technical user. Registration and logging in to the website are not required to access any of the available data or resources. Registration will add a user to the ICTV email list for receipt of newsletters and advanced announcements of webinars and other events. The Drupal theme that provides the template for accessing all data and tools from the website uses responsive web design, so that all pages are accessible and legible on phone and tablet screens.

We have partially implemented [38] and maintain stable, unique IDs for all taxa, as well as release-specific IDs for historical versions of taxonomic records. DOIs for data releases are generated by uploading the MSL and VMR spreadsheets to the data repository Zenodo, with each release assigned a DOI to provide a stable access point for these data (https://zenodo.org/communities/ictv/). We also maintain stable identifiers for the exemplar virus isolate assigned to each species (in the VMR), which are linked to the NCBI accession number of each genomic sequence (and sequence segment for viruses with segmented genomes). All of these ICTV IDs provide stable links to access taxon-specific data (Table 2).

**Table 2.** ICTV Record Identifiers

Identifier	Prefix	Identifies	Link (example)	Comments
MSL	MSL[*release*#].[*version*#]	Taxonomy release	https://ictv.global/id/MSL#(https://ictv.global/id/MSL40)	Master species list (MSL) identifies a specific release of the full taxonomy.
VMR	VMR_MSL[*release*#].[*version*#].*date-of-release*	Virus metadata release	None	Viral metadata resource (VMR) identifies a specific release of the list of virus isolates that are assigned as an exemplar isolate for a given species
Stable taxon	ICTV#	Taxon lineage across releases	https://ictv.global/id/ICTV#(https://ictv.global/id/ICTV20083305)	Identifies a taxon lineage across multiple releases and taxonomic actions (new, rename, move, split, merge, abolish, promote, demote)
Specific taxon	TN#	Specific taxon release	https://ictv.global/id/TN#(https://ictv.global/id/TN19710068)	Identifies a specific taxon in a specific MSL release
Virus exemplar	VMR#	Specific exemplar release	https://ictv.global/id/VMR#(https://ictv.global/id/VMR1001829)	Identifies a specific exemplar isolate in the VMR, linking the GenBank accession(s) for all segments(s) in each isolate

The text and numerical identifiers that specify ICTV taxonomy releases and IDs used to identify current and historical taxa.

The application code, database schema, and data dumps of all database tables that make the data programmatically accessible are available from public GitHub repositories (https://github.com/ICTV-Virus-Knowledgebase).

### Outreach and training

One of the goals of the ICTV is to establish outreach and training programs that introduce and explain the resources of the ICTV website to current and potential users. This is accomplished through help resources provided on the website, webinars, and presentations at scientific meetings.

Instructions for using individual tools and explaining available datasets are provided on the web page of each tool and dataset. Videos explaining how to use the tools and how to find information are available from the Help menu. These how-to videos cover topics such as how to use the tabular Taxonomy Browser, the Visual Taxonomy Browser, and the Find the Species tools. They also cover how to use the MSL and VMR, and the ICTV Report. Information on the history and organization of the ICTV is available from the About menu. User questions, suggestions, and bug reports can be sent via email to the address info@ictv.global. Emails sent to this address are received by the ICTV Data Secretary, who usually provides a response within 24 h.

To provide additional user training, we host webinars focused on using the ICTV website. These webinars have been used to present an overview of the tools and resources available on the website and to demonstrate how to use tools and resources to find information about viruses and their taxonomy. Recordings of all webinars, along with questions from the attendees and answers to those questions, can be accessed under the Help menu of the website. New webinars focused on particular topics related to virus taxonomy (such as submitting taxonomic proposals) or providing instruction on the use of new website or tool features will be offered on a regular basis.

Additional material is available from the Information menu of the website, including EC meeting reports, plenary session reports from the triennial IUMS Virology Congresses, and newsletters.

Finally, to extend its outreach, the ICTV has hosted an exhibit booth at the American Society for Virology meeting over the past several years. At these meetings, all the data, tools, and other resources provided by the website are demonstrated, questions answered, and suggestions for future improvements solicited.

## Future directions

Future enhancements to the ICTV database and web infrastructure will focus on providing additional support for taxonomic classification of viruses, enhancing the user interface and tools for data access and discovery, enhancing the current application programming interface (API), and ensuring the sustainability of the resource into the future. A few specific goals are as follows:

### Support for taxonomic classification

As described above, classification of viruses into taxonomic ranks depends on using taxon-specific demarcation criteria as the basis for determining the correct classification [8, 39]. Currently, these demarcation criteria are defined and published in various locations, including Report chapters, taxonomic proposals, and the published literature. This makes these criteria difficult to find and to determine which source provides the most current information. Therefore, we will be creating a demarcation criteria database to collect and store the pertinent properties and tools used by each individual SG to classify the viruses belonging to the family under their purview. This database will be of significant assistance to the classification of viruses assembled from sequenced metagenomic datasets generated by programs such as the NIH Common Fund-supported Human Virome Program (https://commonfund.nih.gov/humanvirome) and other metagenomic virus discovery sequencing projects [19, 26, 40–43].

### Data access

While the data access features of the ICTV website provide extensive search and display capabilities to support the needs of the typical site user, support for the technical user or support for computational access needs to be improved. Therefore, we will continue to improve, enhance, and extend our API to enable more customized and programmatic access to the ICTV database and tools by data consolidators, public data repositories, and advanced users. This effort will also result in broader alignment with FAIR (findability, accessibility, interoperability, and reusability) principles [38].

### Sustainability

Sustainability of the product of ICTV activities has been a primary goal since the inception of the ICTV in 1966. This is critical given that, as defined by the ICTV Statutes, the membership of the EC changes every three years, and there are strict time limits on how long any individual can serve in each position. Since 1966, the overall membership of the EC has changed 19 times. This provides opportunities for new generations of virologists to participate in and determine the processes used for the creation of the next taxonomy release. But it presents challenges in maintaining the systems and methods used to store and communicate the taxonomy. Therefore, sustainability must be an integral property built into the activities and products of the ICTV and the requirement to communicate its decisions regarding classification and nomenclature and maintain an index (database) of the resulting taxonomy. The resources described in this manuscript represent our initial attempt to fulfill these requirements. The effort expended to maintain a complete history of the virus taxonomy, along with the ability to search and visualize this taxonomic history, also contributes to information sustainability. However, as the size of the taxonomy database grows substantially in the future, and as the resources developed become greater in number and in complexity, the efforts to sustain these resources become even more complex and critical. Therefore, a major goal of our work in the future will be to focus on automation, developing computational pipelines to make it easier to update each individual resource, both when a new or updated taxonomy is published, and as new metadata describing the newly classified viruses is made available. Only in this manner, we will be able to ensure that the 60-year legacy we inherited will be passed on once again to those virologists who will extend it into the future.

## Data Availability

Files containing the current taxonomic data, the MSL and VMR spreadsheets, and all publicly released source code have been assigned DOIs and are available from the ICTV Zenodo data repository (https://zenodo.org/communities/ictv/). Data and source code releases under active development are available from the ICTV GitHub site (https://github.com/ICTV-Virus-Knowledgebase). Unless otherwise noted, all data, source code, and other information provided by the ICTV are provided under the Creative Commons Attribution 4.0 International license (https://creativecommons.org/licenses/by/4.0/).
